# Levels of Physical Activity in Lithuanian Adolescents

**DOI:** 10.3390/medicina54050084

**Published:** 2018-11-12

**Authors:** Guillermo Felipe López-Sánchez, Arūnas Emeljanovas, Brigita Miežienė, Arturo Díaz-Suárez, Sheila Sánchez-Castillo, Lin Yang, Justin Roberts, Lee Smith

**Affiliations:** 1 Faculty of Sports Sciences, University of Murcia, 30720 Murcia, Spain; ardiaz@um.es (A.D.-S.); sheila.sanchez1@um.es (S.S.-C.); 2 Faculty of Sports Education, Lithuanian Sports University, 44221 Kaunas, Lithuania; Arunas.Emeljanovas@lsu.lt (A.E.); Brigita.Mieziene@lsu.lt (B.M.); 3 Department of Epidemiology, Center for Public Health, Medical University of Vienna, 1090 Vienna, Austria; Lin.yang@muv.ac.at; 4 Cambridge Centre for Sport and Exercise Science, Anglia Ruskin University, Cambridge CB5 8DZ, UK; justin.roberts@anglia.ac.uk (J.R.); Lee.Smith@anglia.ac.uk (L.S.)

**Keywords:** physical activity, health, schoolchildren

## Abstract

*Background and objective:* Population levels of physical activity are an international concern. The purpose of the present study was to describe and analyse physical activity levels in Lithuanian adolescents. *Materials and methods:* With this aim in mind, the Physician-based Assessment and Counselling for Exercise (PACE) questionnaire was administered to 5141 adolescents residing in Lithuania, 2502 boys (48.7%) and 2639 girls (51.3%), aged between 11 and 19 years. *Results:* It was found that adolescents studied met the physical activity guideline, of 60 min of moderate-to-vigorous physical activity a day, on average 3.6 days/week (SD = 2.1). A total of 3426 adolescents (66.6%) were inactive as classified by the PACE questionnaire (at least 1 h of physical activity/day < 5 days/week). In the present sample there were more active (at least 1 h of physical activity/day ≥ 5 days/week) boys (n = 994, 39.7%) than girls (n = 721, 27.3%) (*p* < 0.001; OR 1.75, 95% CI 1.56 to 1.97), and, on average, boys were more likely to meet daily recommendations of physical activity than girls, 0.7 days more a week (*p* < 0.001; IRRs 1.21, 95% CI 1.17 to 1.25). According to age, younger adolescents (11–12 years) were significantly more active than older adolescents (13–19 years) and a curvilinear relationship between age and physical activity was observed with significant linear (unstandardized beta (B) = −0.807; standardized beta (β) = −0.796; *p* < 0.001) and quadratic terms (unstandardized beta (B) = 0.024; standardized beta (β) = 0.704; *p* < 0.001). *Conclusions:* It is necessary to increase the level of physical activity in Lithuanian adolescents and intervention programs should be carried out considering these results.

## 1. Introduction

The value of physical activity is fundamental in society for the preservation and development of physical and mental health. Consequently, a physically active lifestyle is related to a healthy lifestyle [1].

Physical activity has been shown to be associated with the improvement of health parameters in children and adolescents, such as physical self-concept, muscular strength, aerobic endurance and favourable blood cholesterol levels [2,3,4,5,6,7], as well as body composition, heart rate variability and sleep quality [8,9,10,11,12]. Nevertheless, despite the health benefits of participating in physical activity, population levels of physical activity in children and adolescents are low [13,14,15,16] and in some countries such as Lithuania schools have been reducing physical activity opportunities (e.g., reducing time allocated to PE) to allow more time for academic pursuits.

In societies with higher socio-cultural development and a high knowledge about the benefits of a healthy lifestyle, there are worse healthy habits, such as sedentary lifestyle or poor-quality diet [15]. The promotion of physical activity and healthy lifestyles is a priority in developed countries [15]. According to guidelines developed by the World Health Organisation (WHO) [17], children and adolescents should do at least one hour of moderate-to-vigorousintensity physical activity per day. The guidelines also state that more than one hour of moderate-to-vigorousintensity physical activity provides greater benefits for health and activity should mainly be of an aerobic nature.

Recent research investigating physical activity levels of children and adolescents indicate that more than 65% do some type of physical activity every week [18,19,20,21,22]. For example, Hernández et al. [20] evaluated the physical activity of 2834 Spanish adolescents and found that 66.2% did physical activity and sport (such as football, basketball, tennis and running) outside the school more than 2 days per week. However, it is evident that the level of physical activity is not enough to meet the guidelines set out by the WHO [19,21,23,24,25,26,27]. Although Lithuania has a low prevalence of overweight and obesity among children and adolescents, from 2005 to 2015, the rates of overweight and obesity have increased from 6% to 16% [8,12]. One plausible explanation is that physical activity levels may be declining in Lithuania and the caloric intake increasing, as it has been suggested in previous studies [8,26].

Janssen et al. and Kalman et al. [8,28] used HBSC (Health Behaviour in School-aged Children, collaborative cross-national study) data and analysed physical activity using the PACE questionnaire in more than 30 countries including Lithuania. These two studies reported a total percentage of active adolescents, without analysing the differences by sex and age. Moreover, the studies of Jansen et al. and Kalman et al. analysed only data from 2001 to 2010 and included data only from adolescents 10-to-16-years-old. Further research is needed in Lithuania to provide an updated description of physical activity among adolescents and compare differences by sex and age.

The aim of the present study was to describe and analyse the current levels of physical activity in a large representative sample of Lithuanian adolescents, by sex and age, considering the percentages of inactive and active adolescents and the average number of days in which they achieve the level of physical activity suggested by the WHO.

## 2. Materials and Methods

### 2.1. Participants and Procedures

Trained research assistants administered questionnaires in randomly-selected Lithuanian public high schools and all the ten counties of Lithuania were included. Two public high schools of each county of Lithuania were invited to participate in the study (total 20 high schools). Private high schools were excluded. An official invitation letter was sent to the 20 high schools randomly-selected for the study and all of them agreed to take part in the study. Also, all adolescents invited agreed to take part in the study. A total of 5141 adolescents (approximately 5% from each school) aged 11–19 years old participated in the study. All subjects gave assent and their parents provided their informed consent for inclusion before they participated in the study. The study was conducted in accordance with the Declaration of Helsinki and the protocol was approved by the Kaunas Regional Biomedical Research Ethics Committee (ethics code Nº BE-2-45; date 9 November 2012). Data were collected from 2013 to 2016.

### 2.2. Instruments

The instrument used was the Physician-based Assessment and Counselling for Exercise (PACE) questionnaire. This questionnaire contains two items (i) the number of days in the last week (PACE 1) the respondent practiced at least one hour of physical activity and (ii) the number of days in a usual week the respondent practiced at least one hour of physical activity (PACE 2). When the compound result obtained from both questions ([PACE 1+PACE 2]/2) was ≥5 days, the person is considered active [23,29]. This questionnaire defines physical activity for the respondents: “*Physical activity is any activity that increases your heart rate and makes you get out of breath some of the time. Physical activity can be done in sports, playing with friends, or walking to school. Some examples of physical activity are running, brisk walking, rollerblading, biking, dancing, skateboarding, swimming, soccer, basketball, football and surfing. Don’t include your physical education or gym class*” [29]. PACE has a test-retest reliability estimated by the Intra-class Correlation Coefficient (ICC) of 0.77 [23,29]. This questionnaire was validated with adolescents in previous studies [23,29]. PACE was firstly validated in American adolescents [29] and afterwards validated also in Spanish adolescents [23]. Double-back translation was performed by a translation team. The questionnaire was translated into Lithuanian, then back to English and the two English versions were compared to check if items had the same meaning. Corrections were made and the final version was administered among the respondents. Respondents completed the questionnaire in the classrooms and anonymously, reporting only basic demographic information (sex and age).

### 2.3. Statistical Analysis

Statistical Package for Social Sciences 23 (SPSS-23) was used for statistical analyses. Descriptive statistics were used to identify frequencies, percentages, means and standard deviations of sample characteristics including physical activity levels. To verify the normality of the distribution of the sample, a histogram of the data was plotted for the variable days a week adolescents participated in at least 60 min of physical activity and both arithmetic means and medians were considered. In addition, mixed binary logistic regression was applied to analyse the differences between active and inactive boys and girls and mixed Poisson regression was applied to analyse the differences in days of physical activity between sexes and ages. In the model, the factors included were sex and school (including school as a random effect) and the covariates age (with linear and quadratic terms). Moreover, unstandardized and standardised effect sizes were calculated. The interaction between sex and age was analysed with Wald test. *p* < 0.05 was considered statistically significant in all cases, using two-sided tests where this is an option.

## 3. Results

A total of 5141 adolescents aged 11–19 years old (mean age 15.4, SD 1.9) took part. 48.7% were boys (n = 2502) and 51.3% were girls (n = 2639). The participation rate was 100%, as all adolescents invited agreed to take part in the study. Participants’ characteristics are detailed in Table 1.

**Figure 1:**
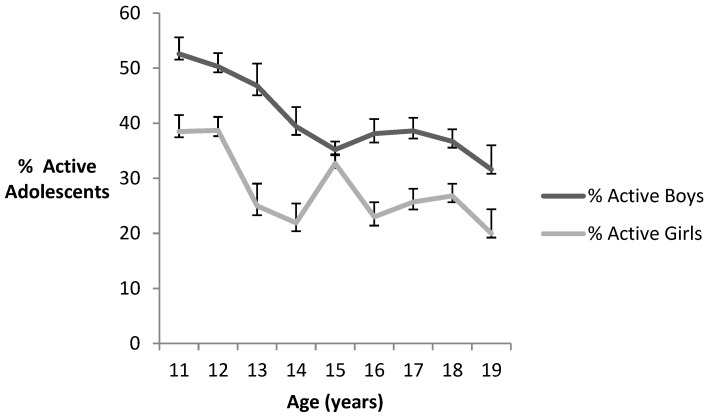
Levels of physical activity.

In Table 2 and Figure 1 frequencies and percentages (%) of participants’ characteristics are presented. Adolescents were classified by level of physical activity (inactive versus active). Table 2 shows that 33.4% of the adolescents studied were classified as active (i.e., meeting daily physical activity guidelines on 5 or more days of the week) and 66.6% of the sample inactive (i.e., not meeting daily physical activity guidelines on 5 or more days of the week). According to sex, the percentage of active boys (39.7%) was higher than the percentage of active girls (27.3%), with a difference of 12.4%. In addition, in all ages there were more active boys than active girls. The differences between boys and girls were significant in all ages, except in the adolescents of 15 and 19 years. Also, a significant interaction between sex and age was found using the Wald test (*p* < 0.001).

**Figure 2:**
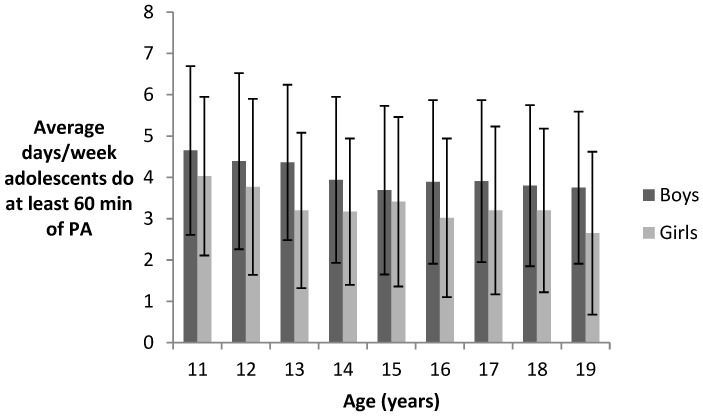
Days/Week adolescents do at least 60 min of PA. Mean (SD).

Table 3 and Figure 2 describe how many days per week (average) the subjects of the sample performed at least 60 min of physical activity, that is to say, the compound result obtained from both questions ([PACE 1+PACE 2]/2). Descriptive statistics are presented according to sex and age. The significant differences between sexes and between ages are also indicated (mixed Poisson regression).

In the whole sample the average number of days per week in which adolescents performed at least the 60 min of daily physical activity recommended by the WHO is 3.60 days. By sex, it can be seen that boys performed more physical activity than girls, in such a way that boys did the needed physical activity an average of 3.96 days per week, whilst girls did it only an average of 3.26 days per week. Therefore, on average boys usually practiced in adequate levels of physical activity 0.7 more days a week than girls. Differences between girls and boys were significant (*p* < 0.05) for the whole sample and for the different ages (except for 11- and 15-year-old adolescents). Also in this case, a significant interaction between sex and age was found using the Wald test (*p* < 0.001).

When the mean values were analysed according to age, it was observed that the lowest levels of physical activity corresponded to 19-year-olds (3.21) and the highest levels of physical activity occurred in 11-year-olds (4.34). Also, with mixed Poisson regression, it was found that the difference between age groups was significant (*p* = 0.001) and there were significant differences between the younger adolescents (11–12 years) and the older adolescents (13–19 years). A curvilinear relationship between age and physical activity was observed with significant linear (unstandardized beta (B) = −0.807; standardized beta (β) = −0.796; *p* < 0.001) and quadratic terms (unstandardized beta (B) = 0.024; standardized beta (β) = 0.704; *p* < 0.001).

## 4. Discussion

The primary aim of this study was to identify levels of physical activity in a large sample of Lithuanian adolescents, comparing the differences by sex and age. In this section, these differences, the possible reasons and possible solutions are discussed.

According to the sample studied, most of the Lithuanian adolescents do not perform enough physical activity, conforming to the guidelines of the WHO. 66.6% of the Lithuanian adolescents studied are inactive. Therefore, only one out of three adolescents do enough physical activity. Boys are significantly more active than girls across all age groups except for those 15 and 19-years. On average, boys perform weekly 0.7 more days of physical activity than girls. Younger adolescents (11–12 years) are significantly more active than older adolescents (13–19 years).

In the present study Lithuanian boys were more active (39.7%) than girls (27.3%), according to the physical activity guidelines [23,29]. Other international studies have found similar findings. For example, Cabak & Woynarowska (2004) [30] observed that boys exhibited higher physical activity than girls in all countries studied. Also, Kalman et al. (2015) [28] studied physical activity in 32 countries from 2002 to 2010 and observed that girls were slightly less likely to show an increase in physical activity over time.

In the same vein, the study of López-Sánchez et al. (2016) [25] with Spanish children and adolescents also found more active boys (31.2%) than active girls (14.9%). Finally, Martínez-Gómez et al. (2009) [23] confirmed this trend too, with adolescents from Madrid: boys practiced physical activity (one hour or more) an average of 3.42 days a week (SD = 1.52) and girls an average of 2.48 days a week (SD = 1.42). Also, the results of the present study showed that Lithuanian boys do physical activity 3.96 days/week (SD = 2.00), while Lithuanian girls do 3.26 days/week (SD = 1.99).

These studies confirm that the differences of physical activity in favour of boys are not only a problem of Lithuania and therefore it is important that international strategies are carried out to promote physical activity in girls, for example through multidisciplinary intervention programs that pay special attention to the promotion of physical activity in girls [27]. The role of mass media and international organizations such as WHO are fundamental to achieve this aim [17,24,25].

According to age, Lithuanian younger adolescents (11–12 years) were significantly more active than Lithuanian older adolescents (13–19 years), although those aged 12 did not significantly differ to those aged 13. This trend is also observed in other international studies. For example, Cabak & Woynarowska (2004) [30], studied Polish youth and found that the percentage of physically active adolescents decreased with age. In the same vein, other studies have found a considerable decline in the levels of physical activity during the period from childhood to adolescence [13,14,15,16].

Nevertheless, this reduction in the level of physical activity from childhood to adolescence is not confirmed in other countries. For example, in some studies carried out in the South of Europe a clear trend of increase or decrease in the level of physical activity according to age is not observed [25]. Therefore, the reduction in the levels of physical activity from younger to older adolescents that has been found in Lithuania and Poland is more common in countries of Northern Europe, possibly related to the lower temperatures that can make more difficult the practice of physical activity. However, it is difficult to know why such differences exist between countries. Although, some possible reasons could be different climates, socio-cultural differences, socio-economic level, or different levels of obesity. Further research is needed to understand such differences. Future international studies should analyse not only the levels of physical activity but also aim to identify country specific barriers and facilitators.

In the specific case of Lithuania, our results suggest that fewer Lithuanian adolescents are physically active when compared to previous studies. For example, Janssen et al. (2005) [8] found that 42.7% of 10- to 16-year-old Lithuanian adolescents were physically active, while in the present study only 33.4%. Also, according to Kalman et al. (2015) [28], who studied the secular trends in moderate-to-vigorous physical activity in 32 countries from 2002 to 2010, with a sample that included 479,674 pupils (49% boys) aged 11 years (n = 156,383), 13 years (n = 163729) and 15 years (n = 159,562), the most significant decreases were reported in Lithuania (boys −11.1%, girls −7.2%).

The results of the present study confirm a decrease in physical activity in Lithuanian adolescents with recent data (2016) and suggests that strategies implemented to increase physical activity in Lithuania are not working yet. These results should be used to design new strategies and interventions to increase levels of physical activity in Lithuanian adolescents, paying special attention to girls and older adolescents. Intervention programs are encouraged from the field of Physical Education but also through physical activity and sport outside the school.

Key strengths of the present study are the large representative sample of Lithuanian adolescents, the participation rate among the adolescents invited (100%) and the use of a validated instrument. The main limitation is that factors such as socio-cultural differences, socio-economic level, schools differences and weight status differences were not analysed. Also, only public schools were considered and therefore our results are not representative of private schools. It should be considered that the questionnaire used excluded physical education/gym classes, as they usually have not enough intensity to be considered beneficial for health and this may overstate the problem of insufficient physical activity. The authors of the present paper recommend future researchers to consider all the factors explained in this paragraph that were not analysed in this study. Furthermore, multidisciplinary intervention programs should be carried out to increase the level of physical activity of Lithuanian adolescents.

## 5. Conclusions

The findings of this study suggest that it is necessary to increase the level of physical activity in Lithuanian adolescents. One possible way to achieve this may be through implementing multidisciplinary programs to reinforce the practice of physical activity. Programs of physical activity may be implemented from the field of Physical Education with the support of local authorities. Moreover, environmental elements such as social and physical environmental factors [31] should be considered when designing and implementing these multidisciplinary programs. Other possible way to increase the level of physical activity in Lithuanian adolescents may be through developing specific movement guidelines for Lithuanian adolescents, as it has been done in Canada [32]. Future researchers and sport and health politicians should take into account all these factors.

## Figures and Tables

**Table 1:** Participants’ characteristics.

Age/Sex	Boys	Girls
All (n = 5141)	2502 (48.7)	2639 (51.3)
11 (n = 233)	116 (49.8)	117(50.2)
12(n = 357)	189(52.9)	168(47.1)
13 (n = 397)	201(50.6)	196(49.4)
14 (n = 437)	218(49.9)	219 (50.1)
15 (n = 896)	449 (50.1)	447 (49.9)
16 (n = 1140)	544 (47.7)	596 (52.3)
17 (n = 924)	415 (44.9)	509 (55.1)
18 (n = 645)	313 (48.5)	332 (51.5)
19 (n = 112)	57 (50.9)	55 (49.1)

Values are Frequency (Percentage).

**Table 2:** Classification by level of physical activity.

Age/Sex	All (n = 5141)		Boys (n = 2502)		Girls (n = 2639)		Sig.	OR (95% CI)
	Active (≥5 Days)	Inactive (<5 Days)	Active (≥5 Days)	Inactive (<5 Days)	Active (≥5 Days)	Inactive (<5 Days)		
All (n = 5141)	1715 (33.4)	3426 (66.6)	994 (39.7)	1508 (60.3)	721 (27.3)	1918 (72.7)	<0.001 *	1.75 (1.56 to 1.97)
11 (n = 233)	106 (45.5)	127 (54.5)	61 (52.6)	55 (47.4)	45 (38.5)	72 (61.5)	0.034 *	1.76 (1.04 to 2.98)
12 (n = 357)	160 (44.8)	197 (55.2)	95 (50.3)	94 (49.7)	65 (38.7)	103 (61.3)	0.039 *	1.57 (1.02 to 2.39)
13 (n = 397)	143 (36.0)	254 (64.0)	94 (46.8)	107 (53.2)	49 (25.0)	147 (75.0)	<0.001 *	2.63 (1.72 to 4.03)
14 (n = 437)	134 (30.7)	303 (69.3)	86 (39.4)	132 (60.6)	48 (21.9)	171 (78.1)	<0.001 *	2.43 (1.59 to 3.71)
15 (n = 896)	304 (33.9)	592 (66.1)	158 (35.2)	291 (64.8)	146 (32.7)	301 (67.3)	0.413	1.12 (0.85 to 1.48)
16 (n = 1140)	344 (30.2)	796 (69.8)	207 (38.1)	337 (61.9)	137 (23.0)	459 (77.0)	<0.001 *	2.06 (1.59 to 2.67)
17 (n = 924)	291 (31.5)	633 (68.5)	160 (38.6)	255 (61.4)	131 (25.7)	378 (74.3)	<0.001 *	1.82 (1.37 to 2.40)
18 (n = 645)	204 (31.6)	441 (68.4)	115 (36.7)	198 (63.3)	89 (26.8)	243 (73.2)	0.006 *	1.59 (1.14 to 2.23)
19 (n = 112)	29 (25.9)	83 (74.1)	18 (31.6)	39 (68.4)	11 (20.0)	44 (80.0)	0.126	1.99 (0.82 to 4.81)

Values are Frequency (Percentage). * Significant differences between sexes. OR: Reference Group: Girls.

**Table 3:** Days a week adolescents do at least 60 min of physical activity.

Age/Sex	All (n = 5141)	Boys (n = 2502)	Girls (n = 2639)	Sig.	IRRs (95% CI)
**All (n = 5141)**	3.60 (2.02)	3.96 (2.00)	3.26 (1.99)	<0.001 *	1.21 (1.17 to 1.25)
**11 (n = 233)**	4.34 (2.00)^13–19^	4.65 (2.04)	4.03 (1.92)	0.062	1.14 (0.99 to 1.31)
**12 (n = 357)**	4.10 (2.15)^14–19^	4.39 (2.13)	3.77 (2.13)	0.031 *	1.15 (1.01 to 1.29)
**13 (n = 397)**	3.79 (1.97)^11^	4.36 (1.88)	3.20 (1.88)	<0.001 *	1.36 (1.22 to 1.52)
**14 (n = 437)**	3.55 (1.93)^11,12^	3.94 (2.01)	3.17 (1.77)	<0.001 *	1.25 (1.11 to 1.39)
**15 (n = 896)**	3.55 (2.05)^11,12^	3.69 (2.04)	3.41 (2.05)	0.072	1.08 (0.99 to 1.18)
**16 (n = 1140)**	3.44 (2.00)^11,12^	3.89 (1.98)	3.02 (1.92)	<0.001 *	1.29 (1.21 to 1.39)
**17 (n = 924)**	3.52 (2.03)^11,12^	3.91 (1.96)	3.20 (2.03)	<0.001 *	1.23 (1.13 to 1.33)
**18 (n = 645)**	3.49 (1.99)^11,12^	3.80 (1.95)	3.20 (1.98)	0.001 *	1.18 (1.07 to 1.31)
**19 (n = 112)**	3.21 (1.97)^11,12^	3.75 (1.84)	2.65 (1.97)	0.006 *	1.45 (1.11 to 1.89)

Values are Mean (SD). * Significant differences between sexes. Significant differences between ages: Indicated by superscripts. IRRs: Reference Group: Girls.
